# General Grant’s Last Days

**Published:** 1885-10

**Authors:** 


### General Grant's Last Days.

From General Adam Badeau's account of the last days of General Grant, in the October *Century*, we quote the following: "On Easter Sunday he seemed a little easier, though there was still no hope. I went into his room and found him able to listen and even to utter a few words without too much effort. I had been greatly struck by the universal watching of a nation, almost of a world, at his bedside, and especially by the sympathy from former rivals and political and even personal adversaries; and I recounted to him instances of this magnanimous forgetfulness of old-time enmities. When I told him of the utterances of General Rosecrans and Jefferson Davis he replied: 'I am very glad to hear this. I would much rather have their good-will than their ill-will. I would rather have the good-will of any man than his ill-will.'

"On the 3d of April several newspapers which had followed General Grant with a persistent animosity down to the very beginning of his illness, recalled in touching and even eloquent words that twenty years before he had captured Richmond on that day. I told this to my chief, for I had been with him on that other 3d of April. I said the nation was looking on now, watching his battle as it did then, and that his fight with disease was as good a one as that he had made with the rebels twenty years before. 'Ah,' he answered, 'twenty years ago I had more to say. I was in command then.' 'But even then,' I replied, 'it took a year to win; perhaps you may win still.' He brightened up at this and told the physicians the story of General Ingalls's dog. Ingalls was the chief quartermaster of the armies operating against Richmond, and had been a classmate with General Grant at West Point; they were always on intimate terms. He had a peculiar dog that often came about the camp-fire at headquarters. One day during the long siege General Grant said, 'Ingalls, do you mean to take that dog into Richmond?' 'I think I shall,' said Ingalls; 'he belongs to a long-lived breed.'

"After this Dr. Shrady sat down to write the bulletin for the morning.

"'What shall I say General?' he asked. 'How shall I tell them you are this morning?'

"'More comfortable,' replied the General.

"And the doctor wrote a line about the physical condition of his patient, and read it to General Grant, who approved. I was still greatly impressed by the public emotion, and I interrupted:

"'General, why not say something about the sympathy of all the world, something to thank the people?'

"'Yes," he exclaimed willingly, and dictated these words: 'I am very much touched and grateful for the sympathy and interest manifested in me by my friends, and by—those who have not hitherto been regarded as friends.'

"Towards the last he stammered and hesitated, evidently unwilling at this moment to call any one an enemy; and finally made use of the circumlocution, 'Those who have not hitherto been regarded as friends.'

"Dr. Shrady wrote out the bulletin, and read it aloud, when the General added: I desire the good-will of all, whether heretofore friends or not.'

"I urged the doctor to stop just there, to say nothing about physical details, but give this Easter message from General Grant to the world in his own language. Mrs. Grant, however, wished the word 'prayerful' to be used before sympathy, and General Grant consented to the change.

"'Another morning, only a day or two after his improvement began, he said to me, evidently with a purpose, that it was strange how undisturbed a man could be when so near death. He supposed he had been as near the other world as one could be and survive. His feeling had been at the time that every moment might be his last; but he had not suffered one particle of apprehension, or fear, or even discomposure. He evidently wished me to know this, for he had once or twice in the winter talked of religious beliefs. 'Yes,' he said, 'at such a time it hurt no one to have lived a good life.' He had been undisturbed--he repeated this emphatically,—but he believed any one would be more comfortable at such a moment with a conscience that could not reproach him. A good life would certainly contribute to composure at the end.

"'The 9th of April came, the anniversary of Appomattox, and recovery was still not assured. One of the sons had a presentiment that his father would not survive that day, but it would have been hard to have General Grant surrender on the anniversary of his greatest victory. Then came another jubilee. His birthday was the 27th of April, and by this time he was so far restored as to be able to join the family for a while at dinner. There were sixty-three lighted candles on the table to celebrate the sixty-three years, which a month before no one had hoped would ever be completed, and the house was crowded with flowers, the gifts of thankful friends. By the 1st of May he was so well that he sent for a stenographer and began to dictate matter for his book. . . .

"The secret of this partial recovery is not far to find. It was after the great expression of public sympathy that General Grant began to improve, after his place in the affections of the people was restored or resumed that his whole nature, moral and physical, became inspired and renovated. For this it was almost worth while to have suffered—to have the world recognize his sensitiveness, and to receive himself its appreciation in return. Few men indeed have known in advance so nearly the verdict of posthumous fame. No death-bed was ever so illumined by the light of universal affection and admiration."

